# Effect of *Lemna minor* supplemented diets on growth, digestive physiology and expression of fatty acids biosynthesis genes of *Cyprinus carpio*

**DOI:** 10.1038/s41598-022-07743-x

**Published:** 2022-03-08

**Authors:** Ravi Kumar Goswami, JaiGopal Sharma, Avanish Kumar Shrivastav, Guddu Kumar, Brett D. Glencross, Douglas R. Tocher, Rina Chakrabarti

**Affiliations:** 1 Aqua Research Lab, Department of Zoology, University of Delhi, Delhi, 110 007 India; 2 Department of Biotechnology, Delhi Technological University, Delhi, 110042 India; 3 Institute of Aquaculture, Faculty of Natural Sciences, University of Stirling, Stirling, FK9 4LA UK; 4 Guangdong Provincial Key Laboratory of Marine Biotechnology, Shantou University, Shantou, 515063 China

**Keywords:** Zoology, Ichthyology

## Abstract

The potential nutritional value of duckweed *Lemna minor* (Lemnaceae) was evaluated for common carp *Cyprinus carpio* fry. Fish were fed diets containing five graded levels of duckweed: 0% (LM0, control), 5% (LM5), 10% (LM10), 15% (LM15) and 20% (LM20). The final weight and specific growth rate were significantly higher in LM15 and LM20 diets fed fish compared to others. Feed conversion ratio was minimum in fish fed diet LM20. Amylase activity was significantly higher in LM0 treatment. Total protease, trypsin and chymotrypsin activities showed linear relationships with the increased level of duckweed in the diet. Protein and essential amino acids contents were significantly higher in carp fed diets LM15 and LM20 compared to others. Lipid content was significantly higher in fish fed duckweed-based diets compared to control. A direct relationship was found between the inclusion level of duckweed in the diet and n-3 long-chain polyunsaturated fatty acid (LC-PUFA) content of carp. Contents of desaturated and elongated products of dietary linolenic acid (18:3n-3) including 20:4n-3, 20:5n-3, 22:5n-3 and 22:6n-3 increased in a graded manner with increasing dietary duckweed. The monounsaturated fatty acids and n-6 PUFA contents reduced significantly in fish fed duckweed. Expression of *fads2d6*, *elovl2*, *elovl5* and *fas* were higher in carp fed diets LM10, LM15 and LM20 compared to control fish. The inclusion of *L. minor* in diet enhanced the nutritional value of carp by increasing protein, lipid, amino acids and n-3 PUFA contents.

## Introduction

Aquaculture is growing faster than other animal food-producing sectors and has raised expectations to bridge the gap between fish demand and supply^1^. The expansion of the aquaculture sector primarily depends on the supply of high-quality feed for cultivated fish. At present, soybean meal is one of the most commonly used alternative ingredients to replace fishmeal in aquafeed due to its high protein content and relatively well-balanced amino acid profile that can generally satisfy the requirements of many fish species^2–5^. Nevertheless, soybean meal is already in high demand in the human food chain both directly and indirectly in feeds for farmed terrestrial animals. This competition means soybean meal is an expensive ingredient and this may limit its use as an ingredient to meet future demands for fish feed. Therefore, there is a constant need to find other ingredients to replace both fishmeal and its main substitute, soybean meal, in feeds for farmed fish. Ideally, such ingredients should be non-conventional to avoid or minimize competition with other animal feed sectors.

The nutritional qualities of feed ingredients including amino acid and fatty acid compositions, micronutrient and fiber contents among others are major considerations when it comes to feed formulation^4–6^. The nutrients must be readily bioavailable to meet the energy and other physiological requirements of the fish and so the overall composition of the ingredient should be appropriate for the digestive enzyme (protease, trypsin, chymotrypsin, lipase etc.) profile of the species, which will help to maximize feed efficiency and production. However, in addition, the composition of feed itself can influence digestive enzyme activities^7^. Thus, feed intake and consumption rates as well as digestive enzyme profiles can be good indicators of health status of fish^8,9^. For plant ingredients, the presence of anti-nutritional factors is one of the potential limitations of their use in aquafeeds^10,11^, although processing, such as fermentation and extrusion can reduce anti-nutritional effects^12–15^. In this overall context, the assessment of aquatic plants as potential ingredients for fish feed is an area of renewed interest and research^16,17^.

Omega-3 (n*-*3) long-chain polyunsaturated fatty acids (LC-PUFA), eicosapentaenoic (EPA, 20:5n-3) and docosahexaenoic (DHA, 22:6n-3) acids are essential dietary nutrients for vertebrates including humans and fish^18,19^. They play important roles in many physiological processes like neural development, immune and inflammatory responses^18^. Primary production of the n-3 LC-PUFA, EPA and DHA, occurs largely in the marine environment and so dietary sources for fish feeds are limited largely to fish oil and fishmeal^20^. In contrast, plant meals and vegetable oils that are the main alternatives to the marine-derived ingredients are completely devoid of EPA and DHA^20^. However, many freshwater fishes are capable of converting the C_18_ PUFA found in plants, linoleic acid (LOA; 18:2n-6) and ALA, to the physiologically essential LC-PUFA arachidonic acid (ARA; 20:4n-6), EPA and DHA^21,22^. Consequently, dietary LOA and ALA can satisfy the essential fatty acid (EFA) requirements of fish species such as common carp and Nile tilapia *Oreochromis niloticus* that have been confirmed to have the metabolic capacity to convert dietary ALA to EPA and DHA^19,23,24^. The conversion of LOA and ALA to LC-PUFA requires a series of fatty acyl desaturase (*fads*) and elongation of very long-chain fatty acids (*elovl*) enzymes such as *elovl2* and *elovl5*^21,22,25,26^*.* Therefore, one potential option for increasing the amount of n-3 LC-PUFA available to human populations is to exploit the endogenous ability of freshwater fish species to produce EPA and DHA from ALA^20^.

The nutritional value of freshwater duckweed *Lemna* spp. is well recognized, having relatively high protein, amino acid and fatty acid contents and low fiber, and so duckweeds can be used as dietary supplements for fish and other animals^16–27^. Specifically, lysine and methionine contents are higher in duckweeds compared to other plants and, therefore, more suitable as ingredients for animal feed^28,29^. *Lemna minor* is also a rich source of the omega-3 (n-3) polyunsaturated fatty acid (PUFA), α-linolenic acid (ALA, 18:3n-3)^16^. Several studies have investigated the use of duckweeds as an ingredient in feeds for carp species, such as *L. minor*^30^ or *L. minuta*^31,32^ in feeds for common carp *Cyprinus carpio*. Furthermore, *L. minor* has been supplemented in diets of rohu *Labeo rohita*^15^, grass carp *Ctenopharyngodon idella* and silver carp *Hypophthalmichthys molitrix*^33^.

Common carp is an omnivorous, bottom-feeding fish belonging to the family Cyprinidae and is one of the most cultured freshwater and economically valued species due to its fast growth and disease resistance^34^. This, combined with the inherent ability for the endogenous biosynthesis of n-3 LC-PUFA, makes common carp an appropriate candidate species to evaluate the utility of duckweed-based diets in freshwater aquaculture.

The overall aim of the present study is to evaluate the duckweed *Lemna minor* as a replacement for plant meals such as soybean meal in extruded pelleted diets for common carp *Cyprinus carpio*. The extrusion process helps to overcome the problems associated with the presence of anti-nutritional factors in *L. minor*. In the present study, the effect of *Lemna minor* supplemented diets on the survival, growth, digestive physiology, and biochemical composition of *Cyprinus carpio* are evaluated. The impact of dietary *L. minor* on the content of EPA and DHA and expression of *fads* and *elovl* genes in fish are also studied. This helps to understand the mechanism of endogenous biosynthesis of LC-PUFA from duckweed-derived ALA in freshwater fish like common carp. The n-3 PUFA content of control diet is very less and it helps to see the effect of *L. minor* supplemented diets on the composition of fish.

## Results

### Survival and growth of fish

After 60 days of culture, the number of fish in each aquarium was counted and no mortalities recorded in any dietary treatment. The final weight of carp fed diets LM15 (2.50 ± 0.012 g) and LM20 (2.66 ± 0.023 g) were significantly higher compared to the fish fed the other diets (Table 1). Final weight was lowest in control diet fed fish (LM0, 1.69 ± 0.005 g) with no inclusion of *L. minor*. The final weight and SGR of fish increased linearly (*p* < 0.05) with the increasing level of *L. minor* in the diet. There was no significant difference in feed intake of fish fed with five different diets. The feed intake of fish ranged from 2.36 to 2.46 g 100 g^−1^ BW day^−1^. The FCR was lowest and highest in carp fed diets LM20 (1.02 ± 0.01) and LM0 (1.27 ± 0.06), respectively. The FCR decreased linearly with the increasing level of duckweeds in the diet of carp.

**Table 1:** Initial and final weights, survival rate, specific growth rate, feed intake, feed conversion ratio and digestive enzyme activities of *Cyprinus carpio* fed with five different diets.

Parameters	LM0	LM5	LM10	LM15	LM20	Polynomial contrasts	
						Linear	Quadratic
Initial body weight (g)	0.48 ± 0.009^a^	0.47 ± 0.008^a^	0.48 ± 0.006^a^	0.48 ± 0.007^a^	0.47 ± 0.005^a^	0.783	0.643
Final weight (g)	1.69 ± 0.005^d^	1.94 ± 0.030^c^	2.10 ± 0.009^b^	2.50 ± 0.012^a^	2.66 ± 0.023^a^	< 0.001	0.425
Specific growth rate (g)	2.11 ± 0.03^c^	2.35 ± 0.05^b^	2.48 ± 0.02^b^	2.76 ± 0.03^a^	2.87 ± 0.01^a^	< 0.001	0.474
Feed intake (g 100 g^−1^ BW day^−1^)	2.36 ± 0.10^a^	2.46 ± 0.12^a^	2.41 ± 0.04^a^	2.44 ± 0.06^a^	2.38 ± 0.02^a^	0.925	0.479
Feed conversion ratio	1.27 ± 0.06^a^	1.22 ± 0.07^ab^	1.14 ± 0.02^b^	1.08 ± 0.02^c^	1.02 ± 0.01^c^	0.001	0.969
Survival (%)	100	100	100	100	100		
**Digestive enzymes**							
Amylase (mU mg^−1^protein min^−1^)	112.44 ± 1.67^a^	75.16 ± 2.06^d^	87.66 ± 0.78^c^	87.22 ± 0.66^c^	104.10 ± 1.14^b^	0.264	< 0.001
Protease (Fluorescence change unit^−1^)	104.31 ± 1.77^d^	107.00 ± 0.53^d^	114.70 ± 1.89^c^	128.80 ± 4.40^b^	151.70 ± 1.23^a^	< 0.001	0.001
Trypsin (µM AMC mg-^1^ protein min^−1^)	822.95 ± 2.10^d^	838.03 ± 4.65^c^	847.41 ± 2.86^c^	1146.73 ± 7.60^b^	1320.30 ± 5.61^a^	< 0.001	< 0.001
Chymotrypsin (µM AMC mg^−1^ protein min^−1^)	1772.69 ± 14.00^d^	1782.77 ± 14.00^d^	2075.77 ± 26.07^c^	2341.07 ± 10.21^a^	2128.35 ± 15.522^b^	< 0.001	< 0.001
Lipase (µM 4-MU mg^−1^ protein min^−1^)	680.98 ± 4.73^b^	731.04 ± 7.55^a^	691.53 ± 6.96^b^	642.67 ± 2.15^c^	626.54 ± 11.84^c^	< 0.001	< 0.001

Values (means ± SE, n = 3) in each row with different superscript are significantly different (*p* < 0.05). The polynomial orthogonal contrast was considered significant at *p* < 0.05 level.

LM0 = Control, soybean; LM5 = 5% *L. minor*; LM10 = 10% *L. minor*; LM15 = 15% *L. minor*; LM20 = 20% *L. minor.*

### Digestive enzyme activities of fish

Amylase activity was significantly lower in carp fed the duckweed-based diets compared to fish fed control diet LM0 (Table 1). The total protease, trypsin and chymotrypsin activities increased linearly (*p* < 0.05) with the increased levels of duckweeds in the diet. Specially, the graded inclusion of dietary duckweed resulted in graded increased activities of protease and trypsin with highest activities recorded in fish fed diet LM20 followed by fish fed diet LM15. Chymotrypsin activity was also higher in fish fed diets LM10–LM20 compared to fish fed the control diet LM0. Lipase activity was significantly higher in fish fed diet LM5 compared to other treatments. The lowest lipase activity was observed in fish fed diet LM20.

### Biochemical composition of fish

The protein and lipid contents of common carp showed linear relationships (*p* < 0.05) with the increased inclusions of duckweeds in the diet (Table 2). Protein content was significantly higher in carp fed diets LM15 (145.7 ± 0.52 g kg^−1^ of fresh weight) and LM20 (148.6 ± 0.57 g kg^−1^ of fresh weight) with the two highest inclusions of duckweed compared to fish fed the other diets. Significantly higher lipid content was observed in fish fed the duckweed-based diets (65.0–67.5 g kg^−1^ of fresh weight) compared to carp fed the control diet without duckweed. The moisture content was significantly higher in fish fed control diet LM0 (771.8 ± 1.24 g kg^−1^ of fresh weight) compared to fish fed the duckweed-based diets. There was no significant difference in ash content among various diets fed carp.

**Table 2:** Biochemical composition of *Cyprinus carpio* cultured under five different feeding regimes (g kg^−1^ of fresh weight).

Parameters	LM0	LM5	LM10	LM15	LM20	Polynomial contrasts	
						Linear	Quadratic
Moisture	771.8 ± 1.24^a^	760.6 ± 0.58^b^	759.6 ± 1.85^b^	757.2 ± 3.10^b^	752.1 ± 4.00^b^	< 0.001	0.285
Crude protein	140.0 ± 0.92^b^	140.8 ± 1.22^b^	142.4 ± 0.85^b^	145.7 ± 0.52^a^	148.6 ± 0.57^a^	< 0.001	0.099
Crude lipid	55.1 ± 1.00^b^	65.0 ± 0.34^a^	65.7 ± 0.62^a^	67.1 ± 0.84^a^	67.5 ± 0.44^a^	< 0.001	< 0.001
Crude ash	20.0 ± 0.01^a^	20.3 ± 0.02^a^	20.9 ± 0.07^a^	21.4 ± 0.03^a^	21.7 ± 0.09^a^	< 0.001	0.749

Values (means ± SE, n = 3) in each row with different superscript are significantly different (*p* < 0.05). The polynomial orthogonal contrast was considered significant at *p* < 0.05 level.

LM0 = Control, soybean; LM5 = 5% *L. minor*; LM10 = 10% *L. minor*; LM15 = 15% *L. minor*; LM20 = 20% *L. minor.*

### Amino acid composition of fish

Total essential amino acid in fish increased linearly with the increased level of duckweed in the diet of carp (Table 3). Arginine, isoleucine and threonine contents were highest in carp fed diet LM20 while histidine, leucine and lysine levels were highest in fish fed the control diet LM0. Phenylalanine content was highest in LM15 diet fed common carp and LM20 followed this group. While there were no significant differences in total non-essential amino acid contents among fish fed the five diets, highest alanine, glycine and serine contents were found in carp fed diet LM20, whereas aspartate, cysteine and glutamic acid levels were highest in fish fed the LM0. Non-proteinogenic amino acids level was lower in fish fed the control LM0 diet compared to fish fed the diets including duckweed. Phosphoserine, taurine, cystathionine, ϒ-amino butyric acid and 1 methyl histidine levels were significantly higher in carp fed diet LM20, while β-alanine and hydroxyproline contents were significantly higher in fish fed diets LM15 and LM20, compared to fish fed the diets with lower duckweed inclusion. In carp fed diets LM0 and LM5, β-amino isobutyric acid was not detected. Like essential amino acids, total non-proteinogenic amino acids level in fish also showed a linear relationship with the increased level of duckweed in the diet.

**Table 3:** Amino acid composition of *Cyprinus carpio* cultured under five different feeding regimes (g kg^−1^ of fresh weight).

Proteinogenic amino acids	LM0	LM5	LM10	LM15	LM20	Polynomial contrasts	
						Linear	Quadratic
**Essential amino acids**							
Arginine (Arg)	6.74 ± 0.13^d^	7.99 ± 0.12^c^	11.79 ± 0.10^a^	10.81 ± 0.11^b^	11.91 ± 0.03^a^	< 0.001	< 0.001
Histidine (His)	3.83 ± 0.01^a^	3.68 ± 0.10^a^	3.29 ± 0.04^b^	3.38 ± 0.03^b^	3.27 ± 0.03^b^	< 0.001	0.018
Isoleucine (Ile)	5.56 ± 0.07^b^	5.46 ± 0.12^b^	4.82 ± 0.07^c^	5.57 ± 0.01^b^	5.97 ± 0.02^a^	0.002	< 0.001
Leucine (Leu)	10.10 ± 0.04^a^	10.05 ± 0.07^a^	8.43 ± 0.04^d^	9.25 ± 0.03^b^	8.89 ± 0.02^c^	< 0.001	< 0.001
Lysine (Lys)	12.53 ± 0.02^a^	11.76 ± 0.11^b^	9.70 ± 0.01^d^	10.24 ± 0.02^c^	10.23 ± 0.03^c^	< 0.001	< 0.001
Methionine (Met)	3.30 ± 0.05^c^	3.37 ± 0.06^c^	3.22 ± 0.06^c^	5.32 ± 0.19^a^	4.08 ± 0.02^b^	< 0.001	0.303
Phenylalanine (Phe)	5.58 ± 0.03^ab^	5.73 ± 0.12^a^	5.42 ± 0.01^b^	5.77 ± 0.02^a^	5.75 ± 0.02^a^	0.082	0.212
Threonine (Thr)	5.24 ± 0.03^d^	5.40 ± 0.08^c^	5.56 ± 0.01^c^	6.19 ± 0.04^b^	6.42 ± 0.08^a^	< 0.001	0.005
Tryptophan (Trp)	1.61 ± 0.04^d^	1.04 ± 0.05^d^	3.55 ± 0.03^a^	2.50 ± 0.08^b^	2.30 ± 0.07^c^	0.007	0.017
Valine (Val)	6.43 ± 0.14^a^	6.45 ± 0.13^a^	5.46 ± 0.70^b^	5.66 ± 0.05^b^	6.72 ± 0.04^a^	0.517	< 0.001
Total	60.92 ± 0.50^b^	60.93 ± 0.30^b^	61.24 ± 0.24^b^	64.69 ± 0.53^a^	65.54 ± 0.49^a^	< 0.001	0.096
**Non-essential amino acids**							
Alanine (Ala)	7.81 ± 0.06^b^	7.11 ± 0.12^d^	7.53 ± 0.06^c^	7.94 ± 0.03^b^	8.25 ± 0.01^a^	< 0.001	< 0.001
Aspartate (Asp)	11.56 ± 0.01^a^	11.39 ± 0.07^a^	10.47 ± 0.07^c^	11.26 ± 0.02^b^	11.06 ± 0.08^b^	< 0.001	< 0.001
Cysteine (Cys)	1.34 ± 0.04^a^	1.48 ± 0.03^a^	1.03 ± 0.03^b^	1.36 ± 0.04^a^	1.14 ± 0.03^b^	0.001	0.718
Glutamic acid (Glu)	20.16 ± 0.04^a^	19.57 ± 0.01^b^	19.30 ± 0.06^b^	18.33 ± 0.15^c^	18.67 ± 0.08^c^	0.001	0.003
Glycine (Gly)	8.29 ± 0.13^d^	9.80 ± 0.08^c^	10.52 ± 0.04^b^	11.16 ± 0.00^a^	11.33 ± 0.11^a^	< 0.001	< 0.001
Proline (Pro)	18.48 ± 0.03^b^	20.45 ± 0.13^a^	18.05 ± 0.09^c^	17.85 ± 0.08^c^	16.73 ± 0.09^d^	< 0.001	< 0.001
Serine (Ser)	4.06 ± 0.05^e^	4.37 ± 0.08^d^	4.66 ± 0.00^c^	5.44 ± 0.03^b^	5.74 ± 0.01^a^	< 0.001	< 0.001
Tyrosine (Tyr)	3.57 ± 0.06^c^	2.61 ± 0.04^e^	4.92 ± 0.01^a^	3.19 ± 0.01^d^	3.85 ± 0.02^b^	< 0.001	0.001
Total	75.27 ± 0.36^a^	76.78 ± 0.20^a^	76.48 ± 0.29^a^	76.53 ± 0.32^a^	76.77 ± 0.38^a^	0.001	0.014
**Non-proteinogenic amino acids**							
Phosphoserine (p-Ser)	0.05 ± 0.00^c^	0.12 ± 0.01^b^	0.11 ± 0.02^b^	0.08 ± 0.01^bc^	0.20 ± 0.03^a^	< 0.001	0.035
Taurine (Tau)	1.69 ± 0.08^b^	1.78 ± 0.03^b^	1.29 ± 0.02^c^	1.80 ± 0.01^b^	2.29 ± 0.02^a^	< 0.001	< 0.001
Cystathionine (Cysthi)	0.42 ± 0.01^b^	0.52 ± 0.02^ab^	0.49 ± 0.04^ab^	0.23 ± 0.02^c^	0.65 ± 0.01^a^	0.244	0.028
β Alanine ( β-Ala)	0.05 ± 0.01^b^	0.06 ± 0.01^b^	0.08 ± 0.05^b^	0.18 ± 0.02^a^	0.19 ± 0.03^a^	< 0.001	0.012
β Amino isobutyric acid ( β -AiBA)	–	–	0.60 ± 0.01^a^	0.37 ± 0.01^b^	0.21 ± 0.02^c^	< 0.001	0.001
ϒ Amino butyric acid ( ϒ—ABA)	0.39 ± 0.06^a^	0.09 ± 0.01^b^	0.02 ± 0.00^c^	–	0.42 ± 0.01^a^	0.497	< 0.001
Hydroxylysine (Hylys)	0.47 ± 0.04^a^	0.41 ± 0.01^a^	0.17 ± 0.01^b^	0.08 ± 0.01^c^	0.03 ± 0.00^d^	< 0.001	0.084
1 Methyl histidine (1 Mehis)	0.09 ± 0.02^c^	0.09 ± 0.00^c^	0.05 ± 0.01^d^	0.19 ± 0.01^b^	0.27 ± 0.01^a^	< 0.001	< 0.001
Hydroxyproline (Hypro)	0.70 ± 0.10^d^	1.23 ± 0.10^c^	1.87 ± 0.07^b^	2.12 ± 0.03^a^	2.15 ± 0.03^a^	< 0.001	< 0.001
Total	3.86 ± 0.16^c^	4.30 ± 0.19^b^	4.68 ± 0.22^b^	4.43 ± 0.08^b^	6.41 ± 0.14^a^	< 0.001	< 0.001
Total amino acids	140.05 ± 0.08^b^	142.01 ± 1.60^b^	142.41 ± 0.21^b^	145.65 ± 0.37^a^	148.72 ± 0.25^a^	< 0.001	0.129

Values (means ± SE, n = 3) in each row with different superscript are significantly different (*p* < 0.05). The polynomial orthogonal contrast was considered significant at *p* < 0.05 level.

LM0 = Control, soybean; LM5 = 5% *L. minor*; LM10 = 10% *L. minor*; LM15 = 15% *L. minor*; LM20 = 20% *L. minor.*

### Fatty acid composition of fish

Total n-3 PUFA content of fish increased with the increasing levels of duckweed in the diets (Table 4). The n-3 fatty acids found in the highest proportions in common carp were eicosatetraenoic acid (ETA 20:4n-3) and DHA, but it was particularly noteworthy that all n-3 PUFA increased in a generally graded manner with the graded increase in dietary duckweed inclusion. Thus, while the increased levels of ALA, the only n-3 PUFA present in the diets, could perhaps be expected, the more interesting result was that the levels of longer, more unsaturated n-3 PUFA including ETA, EPA, docosapentaenoic acid (DPA, 22:5n-3) and DHA also increased significantly with increasing dietary inclusion of *L. minor*. The levels of DPA, DHA and EPA + DHA were all two-fold higher in carp fed diet LM20 with highest duckweed inclusion compared to fish fed control diet LM0 with no duckweed (Table 4). In contrast, n-6 PUFA contents that were dominated by LOA were lower in carp fed the duckweed-based diets compared to fish fed the control diet LM0 and, consequently, the n-6 PUFA: n-3 PUFA ratio decreased from around 6 in carp fed the control diet to 3.8 in fish fed the highest inclusion of duckweed (LM20). However, monounsaturated fatty acids (MUFA), dominated by oleic acid (18:1n-9) regardless of diet, were even more reduced than n-6 PUFA by dietary inclusion of duckweed. Palmitic acid (16:0) was the dominant saturated fatty acid (SFA) in the carp and significantly higher 16:0 and SFA was found in fish fed diet LM5 compared to fish fed the other treatments. Both total SFA and total MUFA decreased linearly with the increased level of duckweed in the diet of carp.

**Table 4:** Fatty acid composition of *Cyprinus carpio* cultured under five different feeding regimes (mg 100 g^−1^ of fresh weight).

Fatty acids	LM0	LM5	LM10	LM15	LM20	Polynomial contrasts	
						Linear	Quadratic
**Saturated fatty acids (SFA)**							
14:0	27.32 ± 0.89^a^	23.61 ± 0.62^b^	17.26 ± 0.41^c^	16.39 ± 0.16^c^	11.39 ± 1.26^d^	< 0.001	0.111
15:0	4.45 ± 0.01^c^	6.10 ± 0.48^b^	5.48 ± 0.98^c^	7.34 ± 0.08^a^	6.39 ± 0.28^b^	< 0.001	0.031
16:0	383.00 ± 0.01^b^	434.12 ± 1.74^a^	354.66 ± 6.64^c^	364.53 ± 5.15^c^	320.44 ± 5.60^d^	< 0.001	< 0.001
18:0	1.90 ± 0.02^c^	4.69 ± 0.20^a^	3.31 ± 0.68^b^	1.38 ± 0.39^c^	3.15 ± 0.45^b^	0.289	0.008
24:0	10.53 ± 0.07^c^	8.49 ± 0.36^d^	11.03 ± 0.09^b^	9.79 ± 0.18^c^	12.89 ± 0.65^a^	< 0.001	< 0.001
Total SFA	427.21 ± 0.89^b^	477.01 ± 1.31^a^	391.74 ± 7.44^c^	399.44 ± 4.78^c^	354.26 ± 7.86^d^	< 0.001	< 0.001
**Monounsaturated fatty acids (MUFA)**							
16:1n-7	26.44 ± 0.01^a^	23.46 ± 0.93^b^	8.21 ± 0.67^c^	4.68 ± 0.41^d^	2.29 ± 0.19^e^	< 0.001	< 0.001
18:1n-9	1014.10 ± 0.40^a^	959.98 ± 0.70^b^	795.25 ± 5.50^c^	745.45 ± 1.29^d^	608.05 ± 6.44^e^	< 0.001	< 0.001
24:1	2.52 ± 0.03^c^	2.55 ± 0.37^c^	3.23 ± 0.05^b^	5.26 ± 0.37^a^	2.78 ± 1.12^bc^	0.010	0.012
Total MUFA	1043.06 ± 0.42^a^	985.99 ± 0.13^b^	806.69 ± 6.19^c^	755.39 ± 1.32^d^	613.12 ± 3.13^e^	< 0.001	< 0.001
**Polyunsaturated fatty acids (PUFA)**							
18:2n-6 (LOA)	1087.98 ± 0.31^a^	948.68 ± 2.69^d^	982.46 ± 9.67^c^	1033.81 ± 8.04^b^	1099.49 ± 0.08^a^	< 0.001	< 0.001
20:2n-6	32.73 ± 1.75^a^	17.43 ± 1.14^c^	24.44 ± 1.47^b^	26.23 ± 3.15^b^	30.66 ± 1.91^ab^	0.230	< 0.001
20:3n-6	16.25 ± 0.92^a^	11.28 ± 1.42^c^	12.42 ± 0.42^bc^	13.92 ± 0.25^b^	10.61 ± 0.17^c^	< 0.001	0.057
20:4n-6	27.41 ± 0.50^a^	22.39 ± 1.12^b^	22.80 ± 0.63^b^	26.65 ± 1.67^a^	21.51 ± 0.25^b^	0.002	0.159
22:5n-6	66.97 ± 1.97^a^	54.07 ± 0.34^b^	49.53 ± 0.13^c^	56.78 ± 0.99^b^	46.22 ± 0.02^d^	< 0.001	< 0.001
Total n-6 PUFA	1231.34 ± 1.52^a^	1053.86 ± 8.75^e^	1091.65 ± 8.85^d^	1157.40 ± 7.20^c^	1208.48 ± 2.42^b^	0.002	< 0.001
18:3n-3 (ALA)	15.22 ± 0.01^c^	14.92 ± 0.69^c^	30.05 ± 0.33^b^	38.35 ± 2.14^a^	38.32 ± 0.78^a^	< 0.001	0.021
20:4n-3	128.04 ± 1.36^d^	166.65 ± 0.40^a^	150.88 ± 4.39^c^	161.30 ± 0.74^ab^	156.96 ± 2.57^bc^	< 0.001	< 0.001
20:5n-3 (EPA)	3.95 ± 0.02^c^	5.01 ± 0.34^b^	5.96 ± 0.05^a^	6.00 ± 0.40^a^	5.28 ± 0.12^b^	< 0.001	< 0.001
22:5n-3	5.39 ± 0.01^d^	9.09 ± 0.35^c^	11.54 ± 0.14^a^	11.10 ± 0.01^ab^	10.42 ± 0.09^b^	< 0.001	< 0.001
22:6n-3 (DHA)	53.60 ± 5.01^e^	81.01 ± 1.77^d^	86.01 ± 0.52^c^	91.03 ± 1.61^b^	107.50 ± 1.68^a^	< 0.001	0.003
Total n-3 PUFA	206.20 ± 6.54^d^	276.68 ± 2.81^c^	284.44 ± 6.02^c^	307.78 ± 1.67^b^	318.48 ± 5.21^a^	< 0.001	< 0.001
EPA + DHA	57.55 ± 5.01^e^	86.02 ± 2.11^d^	91.97 ± 0.58^c^	97.04 ± 1.21^b^	112.79 ± 1.57^a^	< 0.001	0.001
n-6/n-3	5.97 ± 0.03^a^	3.80 ± 0.01^b^	3.84 ± 0.03^b^	3.76 ± 0.02^b^	3.79 ± 0.00^b^	< 0.001	< 0.001

Values (means ± SE, n = 3) in each row with different superscript are significantly different (*p* < 0.05). The polynomial orthogonal contrast was considered significant at *p* < 0.05 level.

LM0 = Control, soybean; LM5 = 5% *L. minor*; LM10 = 10% *L. minor*; LM15 = 15% *L. minor*; LM20 = 20% *L. minor.*

### Gene expression

**Figure 1:**
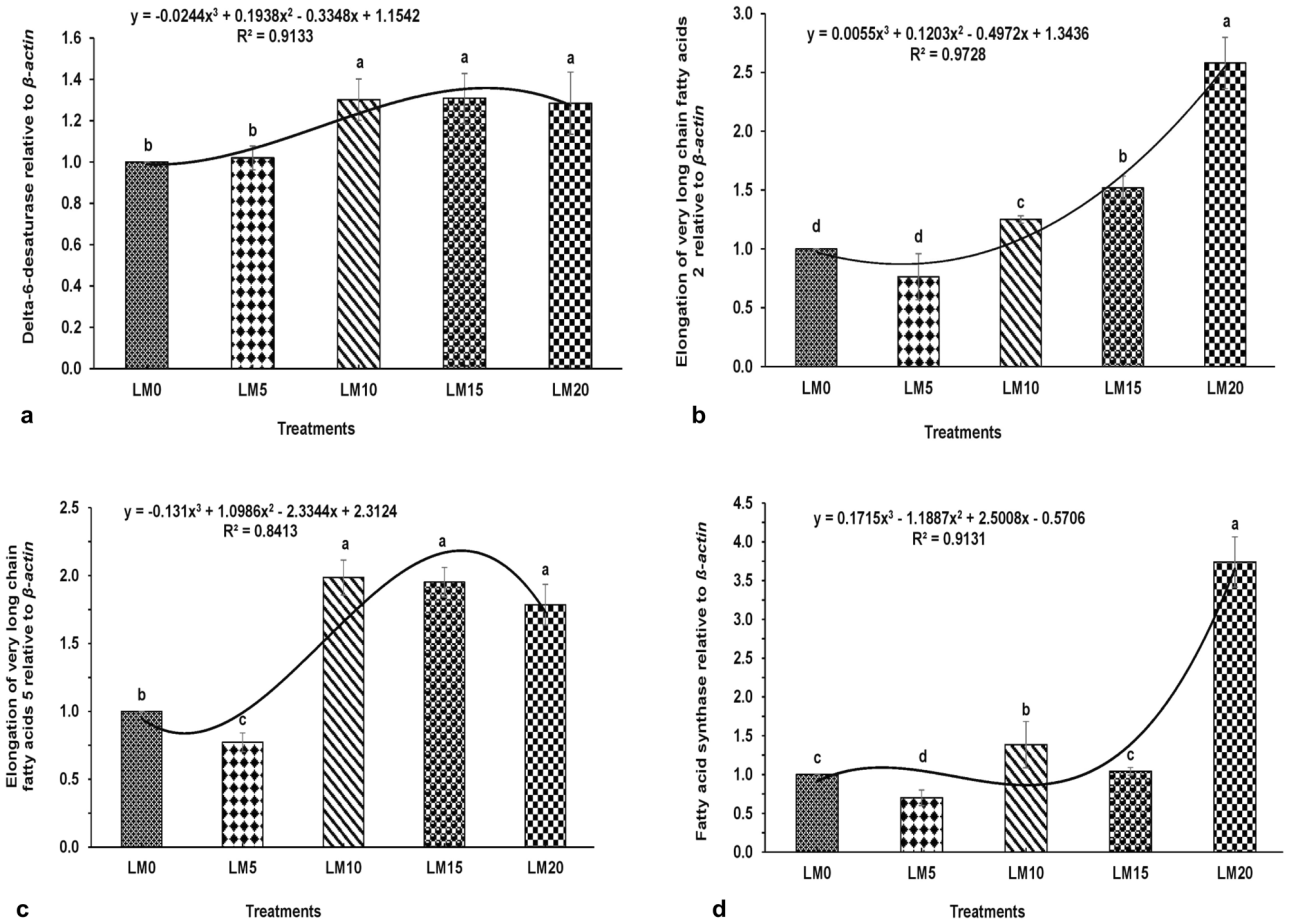
Expressions of (**a**) delta-6-desaturase (*fads2d6)*, (**b**) elongation of very long chain fatty acids protein 2 (*elovl2*), (**c**) elongation of very long chain fatty acids protein 5 (*elovl5*) and (**d**) fatty acid synthase (*fas*) relative to *β-actin* in hepatopancreas of common carp *Cyprinus carpio* cultured under five different feeding regimes. The polynomial (order 3) relationships were found between the diets and the expressions of *fads2d6* (R^2^ = 0.913), *elovl2* (R^2^ = 0.973), *elovl5* (R^2^ = 0.841) and *fas* (R^2^ = 0.913). Bars with different superscripts are significantly (*p* < 0.05; n = 3) different.

The expression levels of delta-6-desaturase (*fads2d6*) was significantly higher in common carp fed diets LM10, LM15 and LM20 compared to fish fed the control LM0 diet (Fig. 1a). The expression of elongation of very long-chain fatty acids protein 2 (*elovl2*) was significantly higher in LM20 diet fed common carp compared to others (Fig. 1b). There was up-regulation of elongation of very long chain fatty acids protein 5 (*elovl5*) in LM10, LM15 and LM20 diets fed fish compared to other group of fish (Fig. 1c). The fatty acid synthase (*fas)* expression was significantly higher in LM20 diet fed common carp compared to others (Fig. 1d). Expression levels of all the genes, except *fads2d6*, were lower in fish fed diet LM5 compared to carp fed the control diet. A polynomial relationship was found between the dietary inclusion levels of duckweed and expressions of various genes.

## Discussion

The duckweed-based diets influenced the growth of common carp in the present study. Final weight and SGR of fish increased linearly as dietary level of duckweed inclusion increased. Similar results were observed in common carp fed diets supplemented with 30–50% *Lemna minuta*^31,32^, grass carp *Ctenopharyngodon idella* fed water hyacinth *Eichhornia crassipes* leaf meal^35^ and rohu *Labeo rohita* fed 20% processed *Pistia* leaves^36^. Feeding of Nile tilapia with fermented *L. minor* supplemented diet (2.5%) enhanced the growth and survival rate of fish^37^. Fresh *L. minor* was supplied with a commercial diet (protein 32%) of juvenile Nile tilapia^38^. Higher final body weight and SGR were found in the experimental diet fed fish compared to the control diet fed one. In the present study, the FCR of carp fed the duckweed-based diets was lower compared to fish fed the control diet suggesting that replacement of soybean meal with duckweed satisfied the nutritional requirements of common carp. Similar trends in FCR were also recorded previously in common carp fed diets including *L. minor* and *L. minuta*^30,32^.

Digestive enzymes play a significant role in the utilization of diets^39^ with the activities of enzymes affecting the efficiency of nutrient absorption, and so their characterization provides key information on the digestive ability of fish to hydrolyze protein, lipid, and carbohydrate in diets^40^. In the present study, amylase activity reduced in carp fed the duckweed-based diets compared to the fish fed the control diet. Similarly, lower amylase activity was observed in rohu fed diets formulated with raw *Pistia* leaves compared to fish fed a reference diet without *Pistia*^36^. However, protein digestion in common carp was influenced by the duckweed-based diets with total protease, trypsin and chymotrypsin activities all significantly higher in fish fed the diets with the two highest levels of duckweed inclusion (LM20 and LM15) compared to fish fed the other diets. Similar results were reported in rohu fed a pelleted diet containing *L. minor*^15^. Higher activities of these enzymes suggested enhanced protein digestion in common carp fed the duckweed-based diets, which indicated that the consumed feed was used more efficiently, resulting in lower FCR and increased growth. Lower protease, trypsin, and chymotrypsin activities were recorded in carp fed the soybean-based diet in the present study. Lower enzyme activities were also observed in Atlantic salmon *Salmo salar*^41^, Nile tilapia^42^ and Japanese seabass *Lateolabrax japonicus*^43^ fed soybean meal-based diets although, in these studies, the reference/control feeds were based on fishmeal. The presence of anti-nutritional factors including protease inhibitors may reduce digestive enzyme activities in these fishes^11,43,44^.

The proximate composition of the carp showed that lipid contents were higher (and moisture lower) in fish fed the duckweed supplemented diets compared to fish fed the control diet. This was consistent with the findings of the previous studies^30,32^ with common carp fed diets containing duckweeds. Higher lipid content was recorded in *I. aquatica* leaf meal and bio-processed *Pistia* leaves supplemented diets fed rohu^36,45^. These data may reflect higher lipid (fatty acid) biosynthesis, as evidenced by the increased expression of fatty acid synthase (*fas*), and tissue lipid deposition. Higher expression of *fas* was observed in tilapia fed palm oil-based diets^46^.

Total amino acids contents (g kg^−1^) would tend to increase in the present study with increasing inclusion of duckweed reflecting the increased protein content in fish fed the highest levels of *L. minor*. The amino acids content varied in different ingredients and thereby, their amount in five different diets. Finally, the composition of diets influenced the amino acid contents of common carp fed with five different diets. The composition of the diets influenced digestibility of the ingested feed. The in vitro digestibility study showed that the digestibility of *L. minor* (6.03 ± 1.78%) was significantly higher compared to soybean meal (3.35 ± 0.01%) in common carp^47^. Higher protein contents were reported in the muscles of *L. minor* supplemented diet fed grass carp and silver carp compared to the soybean supplemented diet fed fishes^33^. Duckweeds exhibit a well-balanced, highly bioavailable source of amino acids for fish^17,28,48^, with essential amino acids such as arginine, histidine, isoleucine, leucine, phenylalanine, threonine, valine, cysteine, and methionine present in relatively high contents in *L. minor*^15,16^. Therefore, duckweeds are regarded as a rich source of essential amino acids that can generally satisfy the amino acid requirements of common carp^5^. In this context, it was noteworthy that carp fed diets LM15 and LM 20 had higher total levels of essential amino acids with arginine, methionine, isoleucine, threonine, tryptophan and valine generally increasing with inclusion of duckweed. Thus, feeding of common carp with duckweed-based diets improved the amino acid composition of the fish.

Fish are an almost unique source of the health beneficial n-3 LC-PUFA, EPA and DHA, for humans^49,50^. Previously, higher levels of EPA, DHA and n*-*3 PUFA were recorded in Nile tilapia fed diets including *Azolla filiculoides*^51^ and duckweeds are a good source of ALA, that is the precursor of n-3 LC-PUFA^16,17,52^. Therefore, in the present study, one focus was to determine if dietary duckweed can boost the levels of n-3 LC-PUFA in carp. The duckweed used in the present study was cultured with the addition of organic fertilizers that can boost lipid content to over 8% of dry weight and ALA content to over 40% of total fatty acids^16^. Furthermore, the inclusion of duckweed in the feeds was at the expense of soybean meal, wheat and corn flours, that are all derived from seeds where n-6 PUFA, specifically 18:2n-6, dominate the fatty acid content. Consequently, increasing inclusion of duckweed increased dietary ALA content by 14-fold while 18:2n-6 PUFA was reduced by around 20% with the n-6 PUFA: n-3 PUFA ratio in the feeds decreasing 17-fold from around 290 to 17. Thus, despite the relatively low lipid content that has precluded aquatic plants like duckweed from being regarded as dietary lipid sources, the inclusion of duckweed had a beneficial impact on the fatty acid composition of the carp feed^16^.

While the increased dietary ALA was also reflected in increased ALA in carp in the present study, more importantly the levels of the n-3 LC-PUFA, ETA, EPA, DPA and DHA also all increased in the carp with the graded increased inclusion of duckweed in the diet. As the feeds were free of any marine ingredients and thus devoid of n-3 LC-PUFA, this result clearly indicated that there was active bioconversion of the dietary ALA, supplied by the duckweed, to DHA in carp resulting in accumulation of n-3 LC-PUFA. This confirmed the earlier findings that freshwater fishes, both herbivorous and omnivorous have the metabolic capacity to convert dietary ALA to EPA and DHA^24,53^. In the present study, the compositional data was supported by the gene expression studies. Several genes including delta-6-desaturase (*fads2d6*), elongation of very long-chain fatty acids protein 2 (*elovl2*) and elongation of very long chain fatty acids protein 5 (*elovl5*) play significant roles in the bioconversion of fatty acids^21,22,54^. Previous studies on the gene expression response of fish after replacing marine ingredients with plant-based diets/vegetable oils showed that the LC-PUFA biosynthesis pathway was stimulated in liver and intestine of various fish species^55–58^. In the present study, the *fads2d6*, *elovl2* and *elovl5* genes were all up-regulated in the hepatopancreas of common carp fed the duckweed diets compared to fish fed the control diet. This was consistent with previous studies that showed a higher level of dietary ALA increased expression levels of *fads2d6* and *elovl5* in common carp^59^, and *fads2d6* and *elovl2* expression levels were up-regulated in juvenile and ongrowing rainbow trout fed plant-based diets^58^. Therefore, the higher levels of EPA + DHA in fish fed the duckweed-based diets confirmed the ability of common carp to synthesize LC-PUFA endogenously from dietary precursor, ALA through increased expression (and activity) of the biosynthetic desaturase and elongase enzymes.

In conclusion, the present study demonstrated that up to 20% duckweed *L. minor* can be included in feeds as a replacement for plant meals including soybean meal without affecting the survival or growth of common carp. The inclusion of duckweed in feeds also enhanced the nutritional value of the carp by increasing protein, lipid, and amino acids levels. Furthermore, duckweed also increased the ALA content of the feed and this enhanced the contents of n-3 LC-PUFA, including EPA and DHA of the carp.

## Materials and methods

### Ingredients and feed formulation

The duckweed *L. minor* was grown in outdoor cement tanks (150 L) fertilized with a mixture of organic manures including cattle manure, poultry wastes and mustard oil-cake as described in detail previously^16^. The duckweed was harvested, washed with clean water, and dried in an oven at 40 °C for 3 h. Other feed ingredients including soybean meal (Ruchi Soya Industries Limited, Mumbai, India), wheat flour (Aashirvaad Atta, ITC Limited, Bangalore, India), corn flour (Ahaar, Private Limited, New Delhi, India), sunflower oil (Fortune, Adani Wilmar Limited, Gujarat, India), amino acids (Himedia, Mumbai, India), vitamin and mineral premixes (Piramal Enterprises Limited, Mumbai) were purchased from the local market. After grinding, ingredients were sieved and stored at 4 °C prior to diet manufacture. The crude protein content of soybean meal, *L. minor*, wheat flour and corn flour were 50.0, 36.07, 12.0 and 12.0%, respectively. The lipid contents of soybean meal, *L. minor*, wheat flour and corn flour were 1.0, 8.45, 1.0 and 1.0%, respectively.

Five marine ingredient-free diets were formulated to contain 32% crude protein and 7% crude lipid using the Winfeed 2.8 software package (WinFeed (UK) Limited, Cambridge, UK) (Table 5). The control diet was based on plant meals with soybean meal as the major protein source (LM0). In four further experimental diets, duckweed was included at increasing levels of 5% (LM5), 10% (LM10), 15% (LM15) and 20% (LM20) of diet dry weight, largely at the expense of soybean meal, wheat flour and sunflower oil to maintain the diets as isonitrogenous, isolipidic and isoenergetic. Earlier study showed that in diet 20% inclusion of *L. minor* affected the survival rate of common carp^30^. Hence, in the present study the maximum inclusion of the duckweed was 20%. The amino acids viz. histidine, methionine, lysine and threonine were added in all diets^5^. All the dry ingredients were blended for 10 min and then mixed with the oil and warm water and feed pellets (1 mm diameter) produced using a BTPL Twin-screw-extruder (Basic Technology Private Limited, Kolkata, India). The pellets extrusion conditions were as follows: cutter rpm 134; feeder rpm 10; extrusion rpm 190; extrusion torque 9.22; heater 1 and heater 2 temperature 65 and 70 °C; final mass temperature 75 °C. The diets were dried at 40 °C before being stored at 4 °C prior to use. The proximate composition study showed that protein, lipid, carbohydrate and ash contents of *L. minor* were 360.70 ± 1.80, 84.50 ± 6.10, 340.70 ± 3.60 and 214.12 ± 2.00 g kg^−1^ (dry weight), respectively^16^. All essential (histidine, isoleucine, leucine, lysine, methionine, phenylalanine, threonine, tryptophan, and valine), non-essential (alanine, arginine, aspartate, cysteine, glutamic acid, glycine, proline, serine and tyrosine) and non-proteinogenic (citrulline, hydroxiproline, taurine etc.) amino acids were found in *L. minor*. The fatty acid composition of *L. minor* was as follows: saturated fatty acids (SFA) 23–26%, monounsaturated fatty acids (MUFA) 11–12%, n-6 polyunsaturated fatty acids (PUFA), mostly linoleic acid (LOA) 17–18% and n-3 PUFA (α-linolenic acid) 41–47%. Based on this finding, the diets were prepared for the present study. The fatty acid compositions of the diets are presented in Table 6 and the amino acid compositions in Supplementary Table 1.

**Table 5:** Ingredients, dietary formulations (g kg^−1^) and analysed proximate compositions of the control and experimental diets.

Ingredients	LM0	LM5	LM10	LM15	LM20		
Soybean meal	500.0	475.0	450.0	425.0	400.0		
*Lemna minor*	–	50.0	100.0	150.0	200.0		
Wheat flour	245.0	223.0	201.0	179.0	158.0		
Corn flour	147.0	147.0	146.0	146.0	146.0		
Sunflower oil	62.0	58.0	55.0	51.0	47.0		
Vitamin/minerals premix^1^	5.0	5.0	5.0	5.0	5.0		
Mono calcium phosphate	20.0	21.0	23.0	24.0	25.0		
Choline chloride	1.0	1.0	1.0	1.0	1.0		
Histidine	1.0	1.0	1.0	1.0	1.0		
Methionine	12.0	12.0	11.0	11.0	10.0		
Lysine	5.0	5.0	5.0	5.0	5.0		
Threonine	2.0	2.0	2.0	2.0	2.0		

						Polynomial contrasts	
						Linear	Quadratic
**Proximate analysis (% dry matter basis)**							
Moisture	6.34 ± 0.08^a^	6.38 ± 0.10^a^	6.20 ± 0.02^a^	6.27 ± 0.04^a^	6.21 ± 0.5^a^	0.089	0.902
Crude protein	31.61 ± 0.14^a^	32.05 ± 0.16^a^	31.42 ± 0.15^a^	31.68 ± 0.21^a^	31.63 ± 0.09^a^	0.285	0.796
Crude lipid	7.42 ± 0.06^a^	7.44 ± 0.12^a^	7.48 ± 0.13^a^	7.35 ± 0.04^a^	7.32 ± 0.05^a^	0.324	0.444
Carbohydrate	47.51 ± 0.08^a^	46.59 ± 0.10^a^	47.19 ± 0.09^a^	47.00 ± 0.09^ab^	46.02 ± 0.07^b^	0.003	0.257
Crude ash	7.22 ± 0.22^a^	7.54 ± 0.07^a^	7.72 ± 0.04^a^	7.70 ± 0.04^a^	7.82 ± 0.08^a^	0.001	0.077
Energy value (Kcal 100 g^−1^)	382.52 ± 0.36^a^	381.52 ± 0.44^a^	381.73 ± 0.58^a^	381.79 ± 0.60^a^	377.38 ± 0.31^a^	0.472	0.075

LM0 = Control, soybean; LM5 = 5% *L. minor*; LM10 = 10% *L. minor*; LM15 = 15% *L. minor*; LM20 = 20% *L. minor.*

^1^Supradyn multivitamin tablets (Piramal Enterprises Ltd., Mumbai, India) containing minerals and trace elements (as mg/kg in diets): = Vitamin A (as acetate) 12; Cholecalciferol 0.1; Thiamine mononitrate, 40; Riboflavine 40; Pyridoxine hydrochloride, 12; Cyanocobalamin, 0.06; Nicotinamide, 400; Calcium pantothenate 65.20; Ascorbic acid 600; α-Tocopheryl acetate,100; Biotin, 1.00. Minerals: Tribasic calcium phosphate, 516; Magnesium oxide, 240; Dried ferrous sulphate, 128.16; Manganse sulphate monohydrate 8.12; Total phosphorus, 103.20. Trace elements: Copper sulphate pentahydrate 13.56; Zinc sulphate, 8.80; Sodium molybdate dihydrate, 1.00; Sodium borate 3.52.

**Table 6:** Fatty acid composition of experimental and control diets (mg 100 g^−1^ of dry weight).

Fatty acids	LM0	LM5	LM10	LM15	LM20	Polynomial contrasts	
						Linear	Quadratic
**Saturated fatty acids (SFA)**							
14:0	10.44 ± 0.07^a^	9.55 ± 0.06^a^	8.82 ± 0.04^a^	10.35 ± 0.20^a^	10.88 ± 2.00^a^	0.361	0.034
15:0	5.20 ± 0.01^a^	4.59 ± 0.17^b^	2.55 ± 0.01^c^	0.81 ± 0.01^d^	0.04 ± 0.00^e^	< 0.001	0.981
16:0	581.01 ± 8.96^a^	531.63 ± 8.38^b^	531.73 ± 1.54^b^	535.31 ± 4.55^b^	538.91 ± 8.96^b^	0.002	< 0.001
18:0	70.09 ± 0.99^a^	64.13 ± 0.93^b^	56.09 ± 0.27^c^	66.21 ± 1.04^b^	40.68 ± 0.51^d^	< 0.001	< 0.001
20:0	3.26 ± 0.36^a^	2.98 ± 0.33^a^	2.44 ± 0.08^ab^	3.12 ± 0.56^a^	1.85 ± 0.25^b^	0.005	0.414
22:0	0.69 ± 0.01^b^	0.75 ± 0.01^b^	0.14 ± 0.00^d^	0.31 ± 0.07^c^	0.90 ± 0.10^a^	0.814	< 0.001
24:0	15.72 ± 2.77^a^	16.77 ± 0.14^a^	9.00 ± 0.46^b^	8.29 ± 0.83^b^	17.25 ± 2.76^a^	0.250	0.001
Total SFA	686.41 ± 6.73^a^	630.40 ± 8.97^b^	610.77 ± 3.09^b^	624.40 ± 3.65^b^	610.51 ± 8.36^b^	< 0.001	< 0.001
**Monounsaturated fatty acids (MUFA)**							
18:1n-9	1460.56 ± 3.81^a^	1336.44 ± 3.95^b^	1163.59 ± 6.70^c^	1163.69 ± 2.02^c^	1059.34 ± 2.20^d^	< 0.001	< 0.001
20:1n-9	7.86 ± 1.24^b^	8.19 ± 0.13^a^	4.09 ± 0.28^c^	3.46 ± 0.33^c^	3.07 ± 0.26^c^	< 0.001	0.369
Total MUFA	1468.42 ± 2.57^a^	1344.63 ± 3.81^b^	1167.68 ± 6.43^c^	1167.15 ± 1.69^c^	1062.41 ± 2.63^d^	< 0.001	< 0.001
**Polyunsaturated fatty acids (PUFA)**							
18:2n-6 (LOA)	3197.60 ± 22.99^a^	2925.76 ± 21.95^b^	2862.71 ± 6.24^b^	2769.39 ± 16.46^c^	2600.36 ± 22.03^d^	< 0.001	0.008
20:2n-6	1.09 ± 0.02^cd^	1.92 ± 0.04^b^	1.38 ± 0.10^c^	0.92 ± 0.12^d^	3.41 ± 0.21^a^	< 0.001	< 0.001
18:3n-3 (ALA)	10.98 ± 1.45^e^	51.10 ± 1.89^d^	81.29 ± 1.35^c^	115.44 ± 0.02^b^	153.10 ± 0.26^a^	< 0.001	0.741
Total PUFA	3209.67 ± 23.42^a^	2978.78 ± 22.80^b^	2945.38 ± 6.50^bc^	2885.75 ± 16.46^c^	2756.87 ± 21.82^d^	< 0.001	0.006
n-6/n-3	291.32 ± 11.23^a^	57.29 ± 2.55^b^	35.23 ± 0.51^c^	23.997 ± 0.21^d^	17.01 ± 0.11^e^	< 0.001	< 0.001

LM0 = Control, soybean; LM5 = 5% *L. minor*; LM10 = 10% *L. minor*; LM15 = 15% *L. minor*; LM20 = 20% *L. minor.*

### Fish culture and feeding

The Institutional Animal Ethics Committee (IAEC) of Delhi University approved the animal care and experimental procedure (DU/ZOOL/IAEC-R/2015/07). The following study was conducted in accordance with relevant guidelines and regulations. The study was carried out compliance with the ARRIVE (Animal Research: Reporting of In Vivo Experiments) guideline.

Fry of common carp were collected from the local fish farm and fed the control diet for 1 week to acclimatize to the experimental conditions. At the initiation of the experiment a total of 450 fry (average weight, 0.47–0.48 g) were distributed randomly among 15 aquaria (30 fry 60 L aquarium^−1^), Each aquarium was fed one of the five diets, LM0, LM5, LM10, LM15 and LM20, for 60 days, with each diet allocated randomly to three aquaria (n = 3). The fish were fed ad libitum to apparent satiation twice per day at 9.00 a.m. and 5.00 p.m. Any uneaten feed was collected after 1 h of feeding to provide an estimation of feed consumption rate. The dissolved oxygen level in the aquarium was maintained by an aerator, and an external filter (Eheim Classic 600, Germany) was employed to reduce ammonia level and maintain water quality of each aquarium. The water temperature (27.8–28.1 °C), pH (7.40–7.62), dissolved oxygen (5.5–5.82 mg L^−1^), ammonia (0.02–0.05 mg L^−1^), nitrate (0.85–1.01 mg L^−1^) and conductivity (380.60–427.03 μS cm^−1^) were regularly monitored in each aquarium using a digital water quality multi-parameter instrument (Hach HQ 40D; Loveland, Colorado, USA). The concentrations of nitrite (0.25–0.48 mg L^−1^) and phosphate (0.08–0.12 mg L^−1^) were measured regularly^60^. There were no significant differences in water quality parameters among the five dietary treatments throughout the study period.

### Sampling

After 60 days of culture, the fish were starved for one day prior to the fish being harvested and euthanized with tricaine methane sulphonate (MS-222; Sigma-Aldrich, USA). The number of fish in each aquarium was counted and recorded to determine survival rate (SR), and the weight of individual fish was measured. The specific growth rate (SGR), feed intake (FI) and feed conversion ratio (FCR) were calculated according to the following formulae.

$${\text{SR}}\;\left( \% \right) = {1}00 \times {\text{final}}\;{\text{number}}\;{\text{of}}\;{\text{fish}}/{\text{initial}}\;{\text{number}}\;{\text{of}}\;{\text{fish}}$$

$${\text{SGR}}\;\left( \% \right) = \left( {{\text{In}}\;{\text{final}}\;{\text{body}}\;{\text{mass}} - {\text{In}}\;{\text{initial}}\;{\text{body}}\;{\text{mass}}} \right) \times {1}00/{\text{duration}}\;{\text{of}}\;{\text{culture}}\;\left( {{\text{days}}} \right)$$

$${\text{FI}}\;\left( {{\text{g}}/{1}00\;{\text{g}}\;{\text{body}}\;{\text{weight}}/{\text{day}}} \right) = {1}00 \times {\text{ total}}\;{\text{feed}}\;{\text{fed}}\;\left( {{\text{dry}}\;{\text{matter}}} \right)/[({\text{initial}}\;{\text{weight}} + {\text{final}}\;{\text{weight}} + {\text{dead}}\;{\text{fish}}\;{\text{weight}})/{2} \times {\text{days}}]$$

$${\text{FCR}} = {\text{feed}}\,\left( {{\text{dry}}\;{\text{weight}}} \right)\;{\text{consumed}}\;{\text{by}}\;{\text{fish}}\;{\text{individually}}\;{\text{during}}\;{\text{feeding}}\;{\text{trial}}/{\text{weight}}\;{\text{gain}}\;\left( {{\text{wet}}\;{\text{weight}}} \right)\;{\text{of}}\;{\text{individual}}\;{\text{fish}}$$

For carcass composition, three fish from each aquarium were collected and pooled, this pooled sample served as one biological replicate (3 replicates per dietary treatment; n = 3). The entire digestive tracts (esophageal sphincter to anus) of another two fish per aquarium were dissected and pooled to provide 3 samples per diet (2 fish per aquarium, 6 fish per diet in 3 replicates; n = 3) for the study of digestive enzymes. The fish were kept in fasting condition for 24 h before sampling and the digestive tract was empty. The hepatopancreas of three individual fish per treatment (1 fish per aquarium, 3 fish per diet; n = 3) were dissected into TRIzol reagent (Ambion, Life Technologies, USA) for the gene expression study. Fish samples (number of fish for assays) were collected following the recommendation of IAEC. All samples were stored at − 80 °C prior to analyses.

### Biochemical compositions of diets and fish

The biochemical compositions of diets and whole fish were determined following the procedures of the Association of Official Analytical Chemists (AOAC) International^61^. Diet samples were ground and the pooled fish samples (three fish) were blended to form a homogeneous paste prior to analyses. Moisture content was determined gravimetrically after drying samples in an oven at 105 °C for 3 h. Ash content was determined after incinerating the oven dried sample in a muffle furnace at 550 °C for 8 h. Crude protein content was determined by the Kjeldahl method (Nitrogen × 6.25; Pelican Instruments, Chennai, India). Crude lipid was determined following the chloroform/methanol extraction method^62^. The subtraction method was used for the determination of carbohydrate contents of feeds^63^, and the energy value of each sample was calculated^64^. All samples were assayed in triplicate.

### Amino acid analysis

Amino acid contents of diets and whole fish were analyzed using a dedicated Hitachi L-8900 High-Speed Amino Acid Analyzer (Hitachi Co. Ltd., Tokyo, Japan). Firstly, samples were hydrolyzed with 6 N HCl at 110 °C for 22 h under N_2_ atmosphere other than tryptophan that was hydrolyzed in a separate sample using 4 N methanesulfonic acid and 3-(2-aminoethyl) indole. Methionine and cysteine were oxidized with performic acid before acid digestion. After digestion, all the samples were evaporated in a Nitrogen Evaporator (PCi Analytic Private Limited, Maharashtra, India) before 0.02 N HCl was added to the dried samples to provide a protein concentration of 0.5 mg mL^−1^ in each sample and 1.5 mL was placed in a glass vial for the autosampler. The amino acids were separated using a cation-exchange resin column (4.6 mm ID × 60 mm L) with 3 μm particle size. The column temperature was 30–70 °C, reaction temperature was 135 °C with a ninhydrin flow rate of 0.35 mL min^−1^. The amino acids were monitored at 570 nm other than proline and hydroxyproline that were monitored at 440 nm. The amino acids were compared with a standard amino acid solution (Wako Pure Chemical Industries Limited, USA). All samples were assayed in triplicate and concentrations expressed as g kg^−1^.

### Fatty acid analysis

The fatty acid compositions of diets and whole fish were measured by gas chromatography (GC). Total lipid was extracted from diet and fish paste samples after homogenization in chloroform: methanol (2:1, v/v) according^62^. Fatty acid methyl esters (FAME) were prepared from total lipid samples by transesterification using 1% sulphuric acid in methanol at 50 °C for 16 h^65^. FAME was separated on a Clarus 580 GC (PerkinElmer, USA) equipped with a ZB-wax column (60 m × 0.32 mm internal diameter × 0.25 μm; Phenomenex, UK). The data were collected from pre-installed programmed software (TotalChrom Workstation Ver6.3). The fatty acids present in diets and fish were identified by comparing the retention times of the sample peaks with a standard fatty acid mixture (Supelco FAME 37 mix, Sigma-Aldrich, USA) and published data^66^. All samples were assayed in triplicate and the concentration was expressed as mg 100 g^−1^.

### Digestive enzyme assays

The samples of whole digestive tract were freeze-dried and homogenized in chilled Milli-Q® water (1:10) to maintain neutral pH of the extracts. The homogenates were centrifuged at 10,000×*g* at 4 °C for 30 min and the tissue supernatants collected for assay of digestive enzyme activities by fluorometric methods (Multimode reader, BioTek Synergy H1 Hybrid, USA). Amylase activity was estimated using Ultra Amylase Assay kit (Invitrogen, USA), pH of the assay mixture was 6.9. The sample was incubated at 25 °C for 25 min. The fluorescence was recorded at 485 nm for excitation and 520 nm for emission. Total protease activity was estimated with EnzChek@ Protease Assay kit (Invitrogen) using phosphate buffer (pH 7.8) at 25 °C. The fluorescence was recorded at 485 nm for excitation and 530 nm for emission. Trypsin activity was estimated following the method^67^ using Tris–HCl buffer (pH 8.0) and Na-benzoyl-l-arginin-methyl-coumarinylamide (Sigma-Aldrich, USA) as substrate. The sample was incubated at 30 °C for 10 min. Chymotrypsin activity was assayed using Tris–HCl buffer (pH, 7.5) and succinyl-Leu-Val-Tyr-4-methyl-coumaryl-7-aminde (Sigma-Aldrich) as substrate^68^. After 10 min of incubation at 37 °C, the fluorescence was recorded at 380 nm for excitation and 450 nm for emission. The substrate 4-methylumbelliferyl butyrate (4-MUB, Sigma-Aldrich) and Tris–HCl buffer (pH, 7.5) were used for the assay of neutral lipase activity^69^. Two sets of same samples were taken and one set was kept at 4 °C (ice bath) and the second one at 37 °C (water bath) for 10 min. The change in fluorescence was recorded at 365 nm for excitation and 450 nm for emission. The value obtained at 4 °C was subtracted from the value obtained at 37 °C. Protein content was estimated following the method using bovine serum albumin (Sigma-Aldrich) as standard^70^. All samples were assayed in triplicate.

### Gene expression analysis

The mRNA expressions of *fads2d6*, *elovl2*, *elovl5* and *fas* genes were studied in the hepatopancreas of common carp. Total RNA was extracted in TRIzol reagent (Ambion, Life Technologies, USA) following the manufacturer's recommendations. The quality of extracted RNA was confirmed using a spectrophotometer (NanoDrop® ND-1000, Thermo Scientific, USA) and integrity of total RNA was determined on 1% agarose gel electrophoresis. The purified RNA (1 µg) was treated with DNase I (Amplification grade 1 kit, Sigma-Aldrich) to eliminate genomic DNA contamination. The DNase-treated RNA was reverse transcribed to cDNA using a High-capacity cDNA Reverse Transcription kit (Applied Biosystems, USA). The cDNA sample for each tank was diluted and used for real-time qPCR to determine gene expression. Quantification of gene expression was performed by Quant Studio 6 Flex system (Applied Biosystems, USA) using PowerUp™ SYBR™ Green Master Mix (Applied Biosystems, USA). Primers were designed using the online primer design tool of NCBI and *β‐actin* was used as the reference gene (Supplementary Table 2). The reaction mixtures for qPCR (10 μL) were composed of 0.25 μL each of forward and reverse primers (2.5 μM), 1 μL of cDNA (1:3), 5 μL of 2X PowerUp™ SYBR™ Green PCR Master Mix and 3.5 μL of RNase-free water. All samples were run in duplicate with each reference gene and negative control (NTC; non template control, containing no cDNA). The cycling conditions of qPCR were as follows: initial activation step at 95 °C for 10 min followed by either 40 cycles of 95 °C for 15 s and 60 °C for 1 min (primer Tm at 60 °C) or 40 cycles of 95 °C for 15 s, 55 °C for 15 s and 72 °C for 1 min (primer Tm < 60 °C). The data of qRT‐PCR were calculated using the 2^−ΔΔC^_T_ method with *β‐actin* as the internal control^71^.

### Statistical analysis

Data were expressed as means ± standard error (SE) with n values as stated. The data were compared using one-way analysis of variance (ANOVA) followed where pertinent by Duncan’s multiple range test, DMR^72^. The polynomial orthogonal contrasts were used to determine the linear and quadratic effect of increasing levels of dietary *L. minor*. The significance level was considered at (*p* < 0.05). All statistical analyses were performed using SPSS software (version 25.0, USA).

### Ethical statement

The Institutional Animal Ethics Committee (IAEC) of Delhi University approved the animal care and experimental procedure (DU/ZOOL/IAEC-R/2015/07). The following study was conducted in accordance with relevant guidelines and regulations. The study was carried out compliance with the ARRIVE (Animal Research: Reporting of In Vivo Experiments) guideline.

## Supplementary Information

Supplementary Information.
