# Supplementary material for: Effect of Lemna minor supplemented diets on growth, digestive physiology and expression of fatty acids biosynthesis genes of Cyprinus carpio
Source: Sci Rep. 2022 Mar 8;12:3711. doi: 10.1038/s41598-022-07743-x (PMC8904519; doi:10.1038/s41598-022-07743-x)
Supplement: Supplementary file 1 — Supplementary Information. [file 41598_2022_7743_MOESM1_ESM.docx]

**Supplementary Table 1.** Amino acid composition of experimental and control diets (g kg^-1^ of dry weight). Data are presented as means ± SE (n = 3).

| **Proteinogenic amino acids** | **LM0** | | **LM5** | | **LM10** | | **LM15** | | **LM20** |
| --- | --- | --- | --- | --- | --- | --- | --- | --- | --- |
| **Essential amino acids** | | | | | | | | | |
| Arginine (Arg) | 17.41 ± 0.20 | | 19.08 ± 0.2 | | 16.60 ± 0.04 | | 19.50 ± 0.20 | | 19.75 ± 0.10 |
| Histidine (His) | 6.98 ± 0.31 | | 8.00 ± 0.05 | | 7.81 ± 0.17 | | 6.91 ± 0.40 | | 6.60 ± 0.12 |
| Isoleucine (Ile) | 12.80 ± 0.20 | | 13.53 ± 0.01 | | 14.25 ± 0.01 | | 13.12 ± 0.07 | | 11.93 ± 0.10 |
| Leucine (Leu) | 22.21 ± 0.32 | | 24.60 ± 0.06 | | 25.67 ± 0.27 | | 23.02 ± 0.30 | | 22.32 ± 0.40 |
| Lysine (Lys) | 20.60 ± 0.10 | | 22.54 ± 0.30 | | 23.60 ± 0.20 | | 22.41 ± 0.01 | | 19.91 ± 0.30 |
| Methionine (Met) | 10.94 ± 0.23 | | 10.88 ± 1.23 | | 13.66 ± 0.50 | | 10.76 ± 0.03 | | 10.81 ± 0.01 |
| Phenylalanine (Phe) | 14.63 ± 0.03 | | 16.14 ± 0.20 | | 15.91 ± 0.30 | | 15.21 ± 0.06 | | 15.06 ± 0.20 |
| Threonine (Thr) | 11.23 ± 0.04 | | 13.80 ± 0.91 | | 13.70 ± 0.14 | | 14.05 ± 0.60 | | 15.96 ± 0.01 |
| Tryptophan (Trp) | 1.11 ± 0.01 | | 1.36 ± 0.03 | | 1.23 ± 0.02 | | 1.16 ± 0.02 | | 1.30 ± 0.20 |
| Valine (Val) | 13.71 ± 0.50 | | 14.90 ± 0.09 | | 15.20 ± 0.70 | | 15.20 ± 0.41 | | 16.31 ± 0.08 |
| **Total** | **131.62 ± 2.20** | | **144.83 ± 2.69** | | **147.63 ± 2.35** | | **141.34 ± 3.60** | | **139.95 ± 1.70** |
| **Non-essential amino acids** | | | | | | | | | |
| Alanine (Ala) | | 12.40 ± 0.01 | | 13.45 ± 0.76 | | 13.59 ± 0.62 | | 12.99 ± 0.20 | 13.49 ± 0.18 |
| Aspartate (Asp) | | 31.31 ± 0.71 | | 34.10 ± 0.50 | | 33.30 ± 0.71 | | 31.14 ± 0.04 | 32.51 ± 0.02 |
| Cysteine (Cys) | | 3.51 ± 0.04 | | 3.41 ± 0.06 | | 2.71 ± 0.04 | | 2.28 ± 0.01 | 2.47 ± 0.03 |
| Glutamic acid (Glu) | | 68.41 ± 5.072 | | 67.13 ± 1.30 | | 65.00 ± 0.38 | | 60.21 ± 1.31 | 63.48 ± 0.20 |
| Glycine (Gly) | | 11.70 ± 0.20 | | 13.01 ± 0.05 | | 13.01 ± 0.60 | | 12.21 ± 0.20 | 12.44 ± 0.07 |
| Proline (Pro) | | 22.46 ± 1.00 | | 19.61 ± 0.11 | | 18.96 ± 0.12 | | 19.61 ± 0.08 | 20.04 ± 0.30 |
| Serine (Ser) | | 13.00 ± 0.33 | | 15.69 ± 0.30 | | 14.80 ± 0.08 | | 14.00 ± 0.34 | 15.16 ± 0.12 |
| Tyrosine (Tyr) | | 9.02 ± 0.20 | | 10.61 ± 0.52 | | 10.40 ± 0.21 | | 8.61 ± 0.40 | 7.97 ± 0.02 |
| **Total** | | **171.81 ± 2.62** | | **177.01 ± 4.90** | | **171.77 ± 3.79** | | **161.05 ± 2.49** | **167.56 ± 1.55** |
| **Non-proteinogenic amino acids** | | | | | | | | | |
| Phosphoserine(p- Ser) | | 1.10 ± 0.31 | | 0.98 ± 0.20 | | 1.26 ± 0.07 | | 0.86 ± 0.01 | 0.81 ± 0.04 |
| Taurine (Tau) | | 0.35 ± 0.00 | | 0.40 ± 0.01 | | 0.36 ± 0.04 | | 0.37 ± 0.00 | 0.17 ± 0.00 |
| Phospho ethanol amine (PEA) | | --- | | 0.67 ± 0.01 | | 0.70 ± 0.01 | | 0.72 ± 0.01 | 0.71 ± 0.03 |
| Cystathionine (Cysthi) | | 1.80 ± 0.70 | | 0.84 ± 0.20 | | 0.87 ± 0.03 | | 0.94 ± 0.10 | 0.59 ± 0.20 |
| β Alanine ( β-Ala) | | 0.20 ± 0.00 | | 0.20 ± 0.01 | | 0.24 ± 0.01 | | 0.18 ± 0.02 | 0.14 ± 0.03 |
| β Amino isobutyric acid ( β -AiBA) | | 0.57 ± 0.50 | | 0.60 ± 0.10 | | 0.55 ± 0.08 | | 0.37 ± 0.01 | 0.26 ± 0.02 |
| ϒ Amino butyric acid ( ϒ- ABA) | | 0.31 ± 0.12 | | 0.32 ± 0.20 | | 0.37 ± 0.17 | | 0.50 ± 0.01 | 0.42 ± 0.01 |
| Hydroxylysine (Hylys) | | 0.10 ± 0.00 | | 0.60 ± 0.00 | | 0.70 ± 0.07 | | 0.80 ± 0.07 | 0.81 ± 0.07 |
| 1 Methyl histidine (1 Mehis) | | 0.58 ± 0.03 | | 0.74 ± 0.05 | | 0.82 ± 0.01 | | 0.78 ± 0.03 | 0.75 ± 0.01 |
| Hydroxyproline (Hypro) | | 0.65 ± 0.12 | | 0.51 ± 0.03 | | 0.70 ± 0.07 | | 2.06 ± 0.04 | 2.39 ± 0.03 |
| **Total** | | **5.66 ± 0.94** | | **5.86 ± 0.80** | | **6.57 ± 0.41** | | **7.58 ± 0.30** | **7.05 ± 0.44** |

**Supplementary Table 2.** Target genes and sequences of primers used for qPCR analysis.

| **Target gene** | **Primer** | **Primer sequence (5’-3’)** | **Accession number** | **Primer efficiency (%)** | **Amplicon size (bp)** |
| --- | --- | --- | --- | --- | --- |
| ***Cyprinus carpio*** | | | | | |
| Delta-6-desaturase (*fads2d6*) | *fads2d6* Fw | AGAAATCCGGAGAAATCTGGCT | AF309557 | 96.01 | 122 |
|  | *fads2d6* Rv | ACTGGCGGTTTAGTTGATGTCT |  |  |  |
| Elongation of very long chain fatty acids 2 (*elovl2*) | *elovl2* Fw | ATCAGTTTGGTCTGCCGGTT | KR706498 | 98.76 | 142 |
|  | *elovl2* Rv | CAGCACAATGAAGATGGTGTCC |  |  |  |
| Elongation of very long chain fatty acids 5 (*elovl5*) | *elovl5* Fw | GATTGACGACACTTCGTCCG | KF924199 | 97.51 | 122 |
|  | *elovl5* Rv | GAAAGTGTGGCTGCAGTGTG |  |  |  |
| Fatty acid synthase (*fas*) | *fas* Fw | AATGCTTGGCAGTCCAGAGT | KY378913 | 96.22 | 139 |
|  | *fas* Rv | AGACACCTGGAACAAGTCCTC |  |  |  |
| *β-actin* | β-actin Fw | AGACATCAGGGTGTCATGGTTGGT | M24113.1 | 99.52 | 352 |
|  | β-actin Rv | CTCAAACATGATCTGTGTCAT |  |  |  |
